# Heterospecific territorial defense in tit species varies according to breeding habitat overlap

**DOI:** 10.1093/beheco/araf082

**Published:** 2025-07-20

**Authors:** Alessandro Berlusconi, Giulia Castiglione, Erminio Clerici, Stefania Martini, Diego Rubolini, Andrea Romano

**Affiliations:** Dipartimento di Scienze e Politiche Ambientali, Università degli Studi di Milano, Via Celoria 26, 20133 Milan, Italy; Parco Pineta di Appiano Gentile e Tradate, Via Alessandro Manzoni, 11, 22070 Castelnuovo Bozzente (CO), Italy; Dipartimento di Scienze e Politiche Ambientali, Università degli Studi di Milano, Via Celoria 26, 20133 Milan, Italy; Conservation Department LIPU-Birdlife Italia, Via Udine 3/a, 43122 Parma, Italy; Dipartimento di Scienze della Vita e Biologia dei Sistemi, Università degli Studi di Torino, Via Accademia Albertina 13, 10123, Turin, Italy; Dipartimento di Scienze e Politiche Ambientali, Università degli Studi di Milano, Via Celoria 26, 20133 Milan, Italy; Dipartimento di Scienze e Politiche Ambientali, Università degli Studi di Milano, Via Celoria 26, 20133 Milan, Italy

**Keywords:** interspecific competition, niche overlap, paridae, playback experiment, territoriality

## Abstract

The coexistence of species within the same guild is promoted by ecological and behavioral mechanisms, particularly niche differentiation. When niches overlap, coexistence may be maintained through spatial segregation, achieved through interspecific territoriality. Most research has focused on pairs of species, with little attention given to complex multispecies guilds. This study investigates the role of interspecific territoriality in promoting the coexistence of 5 sympatric tit species during the breeding season in northern Italy. These species are commonly grouped into “broadleaf” (great tit, blue tit, marsh tit) and “conifer species” (crested tit, coal tit), based on their habitat preferences. Indeed, in the study area, previous observations have shown that their breeding territories are spatially segregated. We experimentally tested whether aggressive territorial behaviors occurred in response to heterospecific playback stimuli, and if they were more intense against heterospecific intruders sharing the same, rather than a different habitat. Our findings revealed that this was the case for all “broadleaf species,” indicating convergent adaptative heterospecific song recognition driven by competition for shared resources. In contrast, “conifer species” did not show such patterns, suggesting possible resource partitioning at a microhabitat scale or differences in breeding territory densities among habitats. This study enhances our understanding of intra-guild interactions and of the mechanisms facilitating coexistence in ecological communities.

## Teaser

How do five similar bird species coexist despite potential competition for space and resources? Our research found that species breeding in broadleaf habitats use vocal signals to aggressively defend territories against other species from the same habitat, reducing direct competition for limited resources. Conversely, species in conifer habitats showed no such behavior, likely relying on resource partitioning. These insights shed light on how competition and resource sharing shape bird communities.

## Background

Territorial behavior is a fundamental behavior allowing resource defense and promoting space partitioning (Verner 1977) . In birds, territorial behaviors involve a combination of direct physical interactions and vocal signal emissions (Newton 1998). The latter are often transmitted over long distances and help mitigating costs associated with overt aggression (Orians and Willson 1964; Grether et al. 2009). Territorial vocalizations, including songs, evolve in response to selection pressures exerted by potential mates and same-sex competitors (Andersson and Iwasa 1996), serving primarily for facilitating mate-acquisition and territorial establishment and defense (Catchpole et al. 2003). Indeed, song traits correlate with fitness, suggesting that songs may convey information about male characteristics to listeners (Kroodsma and Byers 1991). Such song specificity may result in a lack of response to vocalizations from other species (Emlen 1972). The species-specificity of songs is typically achieved through the partitioning of vocalization bandwidths in the acoustic space, which minimizes acoustic masking and reduces interference within a shared “acoustic niche” (Krause 1993). This, in turn, enhances communication efficiency by preventing misinterpretation and overlap with the vocal signals of other species (Villanueva-Rivera 2014).

Yet, some species exhibit interspecific territoriality (Drury et al. 2020), which entails the behavioral exclusion of individuals belonging to other species from a defended space (Maher and Lott 2000). Similar to intraspecific territorial dynamics, interspecific song recognition minimizes the need for engaging in physical aggression (which can lead to severe injuries) or hybridization ( Brambilla et al. 2008; Grether et al. 2009). Interspecific territoriality may emerge as a side effect of interspecific competition, particularly among closely-related species with overlapping ecological niches, ultimately resulting in a stable ecological strategy (Grether et al. 2013). The extent of resource use overlap, together with other factors, including the cost of physical aggression, determines whether natural selection promotes interspecific territoriality (Drury et al. 2020). Hence, this behavior may contribute to spatial niche partitioning, potentially representing an evolutionarily stable alternative (yet not mutually exclusive, see Kirschel et al. 2009) to ecological character displacement to ensure coexistence among similar species (Grether et al. 2009; Drury et al. 2020). Indeed, species demonstrating a higher propensity for interspecific territorial behaviors are those exhibiting high syntopy, similar size, exploiting the same limited resources—like species nesting in secondary tree cavities (Newton 1994) or in environments with limited resources and restricted habitat extent, such as on islands (Reed 1982). Furthermore, interspecific territoriality is typically limited to a few other species only (Doutrelant et al. 2000a; Martin and Dobbs 2014; Freeman 2016). Moreover, these interactions primarily occur in mixed habitats where different species with different habitat preferences may come into contact (Murray 1971; Robinson and Terborgh 1995), resulting to be a facultative context-dependent behavior (McEntee 2014; Drury et al. 2020).

Experimental tests of interspecific territoriality suggest that this behavior is widespread in birds (Mikami and Kawata 2004; Brambilla et al. 2008;  Kirschel et al. 2009; Freeman 2016; Sosa-López et al. 2017; Cowen et al. 2020; Depino and Areta 2020; Jedlikowski et al. 2021). Generally, previous research has shown higher interspecific territorial vocal aggression towards ecologically or genetically similar species compared to control species’ playbacks (Martin and Dobbs 2014; Freeman 2016; Sosa-López et al. 2017). Nonetheless, while interspecific territorial aggression may promote altitudinal vicariance in tropical birds (Jankowski et al. 2010), to date interspecific territoriality among sympatric and cohabiting species has been investigated only in the context of interactions between pairs of interacting species (Hoi et al. 1991; Doutrelant et al. 2000a; Gorissen et al. 2006; Kirschel et al. 2009; Laiolo 2012; Jedlikowski et al. 2021; but see Beckers et al. 2003 for a laboratory study). This may stem from the difficulty of finding conditions where multiple ecologically similar and closely-related species coexist.

Tits (family Paridae) are cavity-nesting songbirds commonly found in woodland habitats of the Northern Hemisphere that exhibit complex social behaviors: during the breeding period, they form stable and strongly territorial pairs, while during winter they usually gather in single- and mixed-species flocks (Herrera 1979). A few previous studies have specifically focused on interspecific aggression and territoriality in pairs of tit species, showing that tits modulate song syntax when co-occurring (Doutrelant et al. 2000a, 2000b; Doutrelant and Lambrechts 2001; Gorissen et al. 2006). A recent study examined the coexistence mechanisms in a guild of five tit species in a sympatric area in Northern Italy composed of the great tit (*Parus major*), blue tit (*Cyanistes caeruleus*), marsh tit (*Poecile palustris*), crested tit (*Lophophanes cristatus*) and coal tit (*Periparus ater*) (Berlusconi et al. 2025). Interspecific comparisons in habitat and space use showed that the guild was composed of two groups: “broadleaf” (ie great tits, blue tits, marsh tits) and “conifer species” (i e crested tits, coal tits), with species within each group using the same set of habitat features and exploiting the same tree canopy sectors to forage during breeding period (Berlusconi et al. 2025). Hence, the key to coexistence may be interspecific spatial segregation, as gauged by the very limited overlap of territory boundaries during breeding (Berlusconi et al. 2025).

The present study aims to experimentally test the hypothesis that interspecific territoriality, manifested as interspecific vocal aggression, promotes spatial segregation of breeding territories among coexisting tit species. We conducted a behavioral experiment using song playback to elicit aggressive territorial responses, to investigate the role of interspecific vocal aggression in interspecific spatial segregation. Specifically, we asked two main questions: 1) do tit species exhibit aggressive behaviors in response to heterospecific playback stimuli within their breeding territories? and 2) are aggressive responses more intense towards heterospecific simulated territorial intrusions of species from the same habitat compared to simulated intrusions of species from different habitats? We predicted the strongest aggressive response to conspecific song stimuli, an intermediate response to heterospecific songs from the same habitat, and the weakest response to heterospecific songs from different habitats. This would align with the hypothesis that greater ecological niche overlap should elicit stronger territorial defense.

## Methods

### Study area and target species

The Pineta Park of Appiano Gentile and Tradate (45°44’ N, 8°56’ E) is a protected area in northern Italy encompassing 4,821 ha, with 73% covered by forests and woodlands, mainly acidophilous conifer forests dominated by Scots pine (*Pinus sylvestris*) and broadleaf trees like chestnut (*Castanea sativa*) in upper plateaus, replaced by mesophilic mixed oak (*Quercus* spp.) and hornbeam (*Carpinus betulus*) forests towards lower altitudes. Additionally, non-native black locust (*Robinia pseudoacacia*) woodlands are present close to farmland areas (Bianchi 2002). This mosaic of coniferous and broadleaved patches creates a rich habitat diversity, harboring abundant populations of 5 breeding tit species (great, blue, marsh, crested and coal tits; Berlusconi et al. 2025). The area thus represented an ideal site to study interspecific competition and territorial behavior among these species.

The 5 tit species are small-sized (10 to 16 cm), differing mostly according to plumage coloration and song type (Hoyo et al. 2007). Great, blue and marsh tits typically live in broadleaf woodlands (Bellamy et al. 2000; Hinsley et al. 2007; Berlusconi et al. 2025), while crested and coal tits inhabit coniferous woodlands (Brotons and Herrando 2003; Berlusconi et al. 2022; 2024). During the breeding period, tits form stable pairs, with males mainly engaging in territorial defense (Cramp and Perrins 1993). In the study area, these species breed mostly synchronously: males occupy and defend breeding territories, mostly by vigorously singing, from mid-March to mid-June, and females lay egg in the same period (see Appendix 1; Table S1).

### Territory mapping

Before conducting playback experiments, we identified and delimited territories of different species across the study area. This was done in four territory mapping areas, characterized by different forest habitat types (see Figure S1 for maps). Observations of territorial individuals were assigned to territories following a modified territory mapping method (Bibby 2000), as outlined in Berlusconi et al. (2024) (see Appendix 2; Figure S1). Territory boundaries were defined based on 7 surveys were conducted in each area between March 16^th^ and April 12^th^, 2023, during territory settlement and pair formation. Territory size was defined based on the minimum convex polygon technique (Hill and Lein 1989; Assandri et al. 2018; Juárez et al. 2020), using “adehabitatHR” package (Calenge 2006). A total of 101 territories were included in the playback experiments (21 territories of great tit, 21 of blue tit, 21 of marsh tit, 20 of crested tit and 18 of coal tit).

### Playback experiment

To investigate territorial aggressiveness, we exposed the identified territorial males to territorial song stimuli produced by a male of the same species (conspecific stimuli), or by heterospecific males sharing the same main breeding habitat as the focal species (heterospecific intra-habitat stimuli), or by heterospecific males occupying a different breeding habitat to the focal species (heterospecific extra-habitat stimuli). Each territorial male received (at least) 1 stimulus for conspecific treatment, and 2 randomly selected stimuli for heterospecific (intra- or extra-habitat) treatments. In some randomly selected cases (N = 22), territorial males were exposed to a maximum of 4 stimuli (1 conspecific + 3 randomly selected heterospecific). The order of conspecific and heterospecific (intra- or extra-habitat) stimuli was randomized within males thus obtaining differences among males. The selection of the heterospecific (intra- or extra-habitat) playback stimulus for each territorial male was randomized while ensuring that males of each species were exposed to minimum of 8 heterospecific stimuli of each other species. Across all 5 species, we balanced playback treatments to maximize comparability while accounting for field logistics and species availability. We performed a total of 333 trials, of which 103 were conspecific, 97 heterospecific intra-habitat and 133 heterospecific extra-habitat. For each focal species, the Table 1 reports how many trials involved playbacks of each possible species,

**Table 1.** Treatment combination matrix, which shows trial combinations between species playback and focal male species. The table provides the number of conspecific trials (in which we exposed the territorial males to territorial song stimuli produced by a male of the same species), correspondinig to the number of males tested, the number of “intra-habitat” heterospecific trials (territorial males exposed to other species stimuli from the same habitat of the focal species) and the number of “extra-habitat” heterospecific trials (territorial males exposed to other species stimuli from different habitat of the focal species).

		Playback species				
		Great tit	Blue tit	Marsh tit	Crested tit	Coal tit
**Focal species**	**Great tit**	21	12	11	12	14
	**Blue tit**	14	21	12	9	12
	**Marsh tit**	11	12	23	15	10
	**Crested tit**	11	11	12	20	14
	**Coal tit**	9	10	8	11	18

A playback trial started by placing a speaker (JBL omnidirectional speaker, Model Charge 5, frequency response between 60 Hz and 20 kHz) 10 m from the observers, near the center of the territory, hanging the speaker from a pole 4 m above the ground. The speaker was placed in the same location within the male territory for all playback trials. Flagging tape was placed on two sides of the speaker at a distance of 4 m to assist in distance estimation. In all cases, the observer estimated distance and heights to the nearest meter. Each playback trial lasted 9 min (1 min observation period, no playback; 4 min playback stimulus broadcasting; 4 min observation period). If a bird was already engaged in aggressive interactions with a rival at the beginning of the trial, the trial was discarded to avoid confounding effects of overstimulation (see also Doutrelant et al. 2000a; Gorissen et al. 2006; Freeman 2016). We assessed the response of territorial males during the playback broadcast and in the following observation period (Sosa-López et al. 2017).

The song stimulus was broadcast at the natural species-specific sound pressure level, which ranges from 80 to 95 dB at 1 m depending on the specific phrase emitted by each species (previously recorded and measured). Songs used for playback stimuli were sourced from the Xeno-canto database (Planqué and Vellinga 2003) and selected exclusively from recordings made in Northern Italy, ensuring they belonged to populations of the same geographic area. For each species, playback tracks were created using three different recordings from three different individuals to enhance representativeness. For each playback session, the order of these 3 tracks was randomized to avoid order effects (Kroodsma 1989) . The only manipulation applied was volume adjustment to standardize playback intensity across all species while preserving natural variation in song structure and phrasing.

During preliminary trials, fifteen randomly selected territorial males (three from each tit species) were exposed a further control stimulus, the song of the common chaffinch (*Fringilla coelebs*). However, no territorial response to chaffinch song was observed in any of the tested males.

Playback trials were conducted with favorable weather (eg no wind or rain). All trials were performed in the early morning (between 06:00 am and 10:30 am GMT+1) from March 29^th^ to April 28^th^, 2023. This represents the period of highest responsiveness towards conspecific songs in our study populations (authors’ pers. obs.) and it aligns with prior studies of populations of the same species breeding at comparable latitudes (Doutrelant et al. 2000a). To avoid overstimulation, playback experiments were performed for each territorial male only once per day and were separated by at least 2 d (mean value ± S.D.: 4.56 ± 2.45 d); furthermore, males defending adjacent territories were never stimulated during the same day.

### Quantifying territorial male responses to playback

During each trial, focal males were sampled by continuous focal observations (Altmann 1974) with behavioral data recorded every second throughout the observation period. As soon as the playback started, one operator continuously observed the behavior of the territorial male with 8-10 × 42 binoculars 10 m from the speaker, dictating the information to a second operator who compiled an ethogram. We also audio-recorded the session for additional verifications.

Specifically, we recorded the focal male’s distance to the speaker (in meters) and the time spent singing (in seconds), noting these values every second for the duration of the observation period; we then calculated a set of variables typically associated to the aggressive behavioral responses to an intruder or to vocal stimuli according to literature (Martin et al. 1996; Jankowski et al. 2010; Martin and Dobbs 2014; Freeman 2016; Sosa-López et al. 2017): minimum distance to the speaker (m), mean distance to the speaker during singing (m), total time spent singing (s), latency of the vocal response to the playback (s). These variables were weakly or only moderately correlated (Pearson’s *r* < 0.60). Each variable was converted to 0-1 scale, where 0 represented the weakest aggressive response and 1 the strongest one. Specifically, total time spent singing was directly scaled from 0 to 1, as it has a direct relationship with aggression. In contrast, for minimum distance to the speaker, mean distance to the speaker during singing and latency of the response, which have an inverse relationship with aggression, we first scaled the values from 0 to 1 and then applied a transformation (1—scaled value) to ensure consistency in scoring. For example, concerning the total time spent in singing, if an individual sang for 120 s out of 480 s of observation, it was assigned a value of 0.25 (i.e. 120/480). These values were then summed to calculate an overall continuous score, ranging from 0 to 4. Subsequently, all scores for a given territorial male were centered to account for individual variability, ensuring the values reflected the within-individual differences in response to different playback stimuli. This approach resulted in an “aggression index” that was centered per individual, normally distributed, with a mean around 0. Positive values of the aggression index indicated trials when a given territorial male behaved more aggressively than its average aggressiveness level, while the opposite held true for negative values. We preferred to summarize aggressive behavioral responses using an “aggression index” rather than a Principal Component Analysis (PCA) for both biological and statistical reasons. Centering the index around each male’s mean allowed us to capture within-individual variation in response to playback, isolating changes relative to each bird’s average level of aggression. This approach also produced a normally distributed variable with mean zero, well-suited for modeling. In contrast, PCA ignores individual-level variability and assumes similar covariation among alle 4 behavioral variables across species—an assumption not supported in our system, where species differ in how they express aggression. Moreover, PCA components lack a consistent biological interpretation, as loadings depend on dataset structure.

We also recorded if the territorial male was singing before playback broadcasting (without directly interacting with a rival), because this condition could result in a stronger aggressive response.

### Statistical analyses

We firstly tested whether a difference in aggressive behavior in response to different playbacks emerged between species living in different habitats, relying on a linear mixed model (LMM), fitted using the package “glmmTMB” (Brooks et al. 2017) in R 4.0.3 (R Core Team 2022). We included data for all species to examine the effect of playback treatment (3-level factor: conspecific, intra-habitat heterospecific, extra-habitat heterospecific), species group (2-level factor: “broadleaf” species, “conifer” species) and their interaction as fixed effects, while controlling for male singing (2-level factor: no = 0, yes = 1; it accounts for the fact that a male was already singing or not at the beginning of the trial), trial sequence (progressive trial number for that territorial male, in order to assess any positive, reflecting exacerbation, or negative, reflecting habituation, on response to playback), and the time of day (min after sunrise; to control for variation in daytime activity). Continuous predictors were standardized (mean = 0 and SD = 1) before fitting models. The quadratic effect of time of day was also tested in exploratory analysis, as well as the interaction between playback treatment and male singing, but were invariably statistically non-significant (all *P* > 0.05) and therefore were not included in final models (details not shown for brevity). Territorial male identity (N = 101; see Table 1  for species-specific sample size) was included as a random intercept, nested within target species (N = 5), while playback species (N = 5) was also included as a further random effect.

Thus, to investigate variation in patterns of interspecific aggressiveness, we relied on a series of LMMs, fitted separately for each species. All models had the same structure, including the aggression index as response variable, and the following fixed effect predictors: the playback treatment (3-level factor: conspecific, intra-habitat heterospecific, extra-habitat heterospecific), male singing (2-level factor: no = 0, yes = 1), trial sequence, and the time of day. Continuous predictors were standardized, and quadratic effect of time of day was also tested in exploratory analysis. Territorial male identity and playback species were entered as random intercept terms to account for non-independence of experiments performed in the same territory and for the same playback species. Significance of parameter estimates was tested using likelihood ratio tests (Singmann et al. 2015). Post hoc tests among levels of playback treatment were also performed using the R package “emmeans” (Lenth 2017)  with P-values for each variable combination corrected according to False Discovery Rate. Model assumptions were checked by inspection of model outputs via the R package “performance” (Lüdecke et al. 2021).

We also tested whether responses to playback were species-specific. To this end, we fitted different LMMs for each of the four aggression variables: minimum distance to the speaker, mean distance to the speaker while singing, total time spent singing, and latency of the vocal response. To account for individual-level variation, all response variables were centered within each territorial male, following the same procedure used for the aggression index described above. In all models, playback treatment was included as a fixed effect, together with male singing and time of day. Territorial male identity and playback species were entered as random intercept terms. Post hoc tests among levels of playback treatment were also performed with P-values for each variable combination corrected according to False Discovery Rate.

We also examined whether interspecific response was influenced by differences in body size and song features among pairs of competing species. After obtaining mean aggression indexes for each focal and playback species combination, we assessed their correlation with body mass differences and acoustic similarity. The detailed methodology for these analyses is provided in Appendices 3 and 4. Briefly, we gathered body mass data from the ANITA databank (Italian Bird Ringing Centre, ISPRA) and calculated pairwise species differences to build a body size difference matrix. Similarly, we used Dynamic Time Warping (DTW) analysis on playback stimuli to quantify song similarity relying on the alignment of syllables, using several the acoustic features, thus generating an acoustic similarity matrix. We then relied on a permutation-based approach to test the relationship between these matrices and interspecific aggression.

## Results

The model fitted on the entire dataset highlighted that species living in different habitats responded differently to the playback treatments (Table 2).

**Table 2.** Linear Mixed Model testing for variation in the aggression index (centered within each territorial male) according to playback treatment (conspecific, intra-habitat heterospecific, extra-habitat heterospecific) in interaction with habitat subgroup (“broadleaf” vs. “conifer” species). Additionally, we included whether the territorial male was already singing, the progressive trial number, and the minutes after sunrise as fixed effects. Territorial male identity (N = 101) was included as a random intercept nested within Species (N = 5), while Playback species (N = 5) was also entered as a random intercept term. Statistically significant predictors (*P* < 0.05) are reported in bold. Marginal R^2^ values are also shown. Total number of records = 333.

Predictors	Coefficient ± S.E.	*χ^²^*	df	*P*
**Playback treatment**	**-**	**80.33**	**2**	**<0.001**
Habitat subgroup	-	3.83	1	0.050
**Playback treatment × Habitat subgroup**	-	**12.43**	**2**	**0.002**
Already singing	0.37 ± 0.10	13.53	1	<0.001
Prog. trial	-0.05 ± 0.05	1.18	1	0.277
Minutes after sunrise	0.02 ± 0.04	0.31	1	0.579
*Marginal R* ^*2*^ * = 0.40*				

**Fig. 1.**
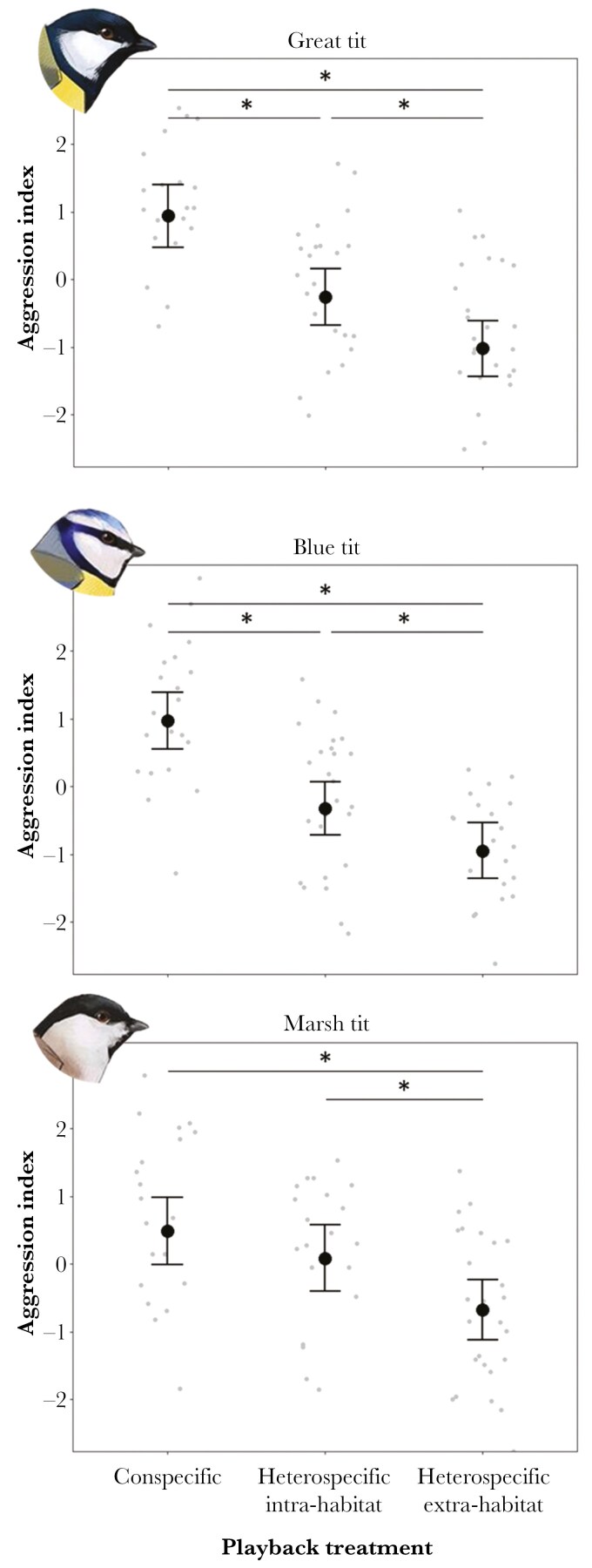
Variation in aggression index for the “broadleaf species” (i.e.: great tit, blue tit, and marsh tit) according to playback treatments (conspecific, heterospecific intra-habitat, and heterospecific extra-habitat). Gray dots represent aggression index centered values within each territorial male. Black dots show model-predicted means from Linear Mixed Models from Table 3 , with whiskers indicating standard errors. Asterisks denote statistically significant differences between treatments in post hoc tests (*P* < 0.05) – details in Table 3.

**Fig. 2.**
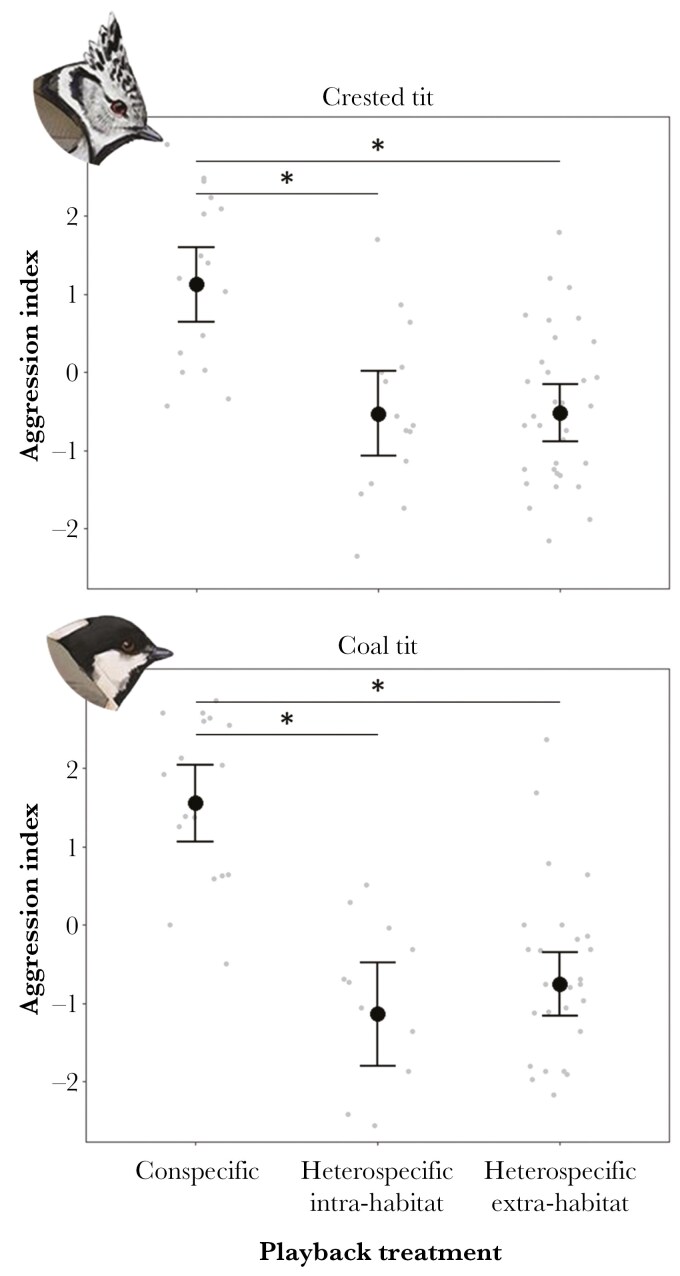
Variation in aggression index for the “conifer species” (i.e.: crested tit and coal tit) according to playback treatments (conspecific, heterospecific intra-habitat, and heterospecific extra-habitat). Gray dots represent aggression index centered values within each territorial male. Black dots show model-predicted means from Linear Mixed Models from Table 3 , with whiskers indicating standard errors. Asterisks denote statistically significant differences between treatments in post hoc tests (*P* < 0.05) – details in Table 3.

Aggression index significantly varied among playback treatments in all species, the response to conspecific playback being generally more aggressive than the response to the heterospecific stimuli, except for the marsh tit, where there was no difference in aggressive response between conspecific and heterospecific intra-habitat stimuli (Table 3, Figs. 1, 2; see also Table S3, S4, Figure S2-S7 for species-specific responses). Moreover, within the “broadleaf species” group (i.e. great tits, blue tits, and marsh tits), aggressiveness was greater for intra-habitat heterospecific stimuli compared to extra-habitat ones (Table 3, Fig. 1). However, no significant differences in the response to intra-habitat and extra-habitat heterospecific stimuli were observed in the crested and the coal tit (Table 3, Fig. 2). Great and blue tit males that were singing at the beginning of playback trials showed significantly increased aggressiveness compared to those that were not, while this was not the case for the other species (Table 3). Trial sequence and time of day did not significantly predict aggressiveness (Table 3).

**Table 3.** Linear Mixed Models testing for variation in the aggression index (centered within each territorial male) of great tits, blue tits, marsh tits, crested tits and coal tits according to the playback treatment (conspecific, heterospecific intra-habitat, heterospecific extra-habitat), male singing, trial sequence and time of day. Territorial male identity (N = 101) and playback species (N = 5) were entered as random intercept terms. Since the playback treatment was statistically significant in each species, post hoc tests were conducted to examine pairwise comparisons between the levels. Marginal R^2^ values are also shown.

Predictors	Coefficient ± S.E.	*χ* ^^*2*^^ */ t*	df	*P*
*Great tit (N = 70, marginal R^2^ = 0.45)*				
**Playback treatment**	**-**	**42.01**	**2**	**< 0.001**
** Conspecific vs heterospecific intra-habitat**	**1.20 ± 0.29**	**4.19**	**59**	**0.001**
** Conspecific vs heterospecific extra-habitat**	**1.96 ± 0.30**	**6.41**	**59**	**< 0.001**
** Heterospecific intra-habitat vs heterospecific extra-habitat**	**0.76 ± 0.29**	**2.64**	**59**	**0.010**
**Already singing**	**0.56 ± 0.27**	**4.26**	**1**	**0.039**
Prog. trial	0.12 ± 0.13	0.92	1	0.338
Minutes after sunrise	0.10 ± 0.12	0.74	1	0.391

*Blue tit (N = 68, marginal R^2^ = 0.46)*				
**Playback treatment**	**-**	**43.37**	**2**	**< 0.001**
** Conspecific vs heterospecific intra-habitat**	**1.29 ± 0.28**	**4.54**	**57**	**< 0.001**
** Conspecific vs heterospecific extra-habitat**	**1.92 ± 0.30**	**6.49**	**57**	**< 0.001**
** Heterospecific intra-habitat vs heterospecific extra-habitat**	**0.63 ± 0.27**	**2.32**	**57**	**0.024**
**Already singing**	**0.61 ± 0.28**	**4.68**	**1**	**0.031**
Prog. trial	-0.06 ± 0.12	0.29	1	0.587
Minutes after sunrise	0.03 ± 0.12	0.05	1	0.817

*Marsh tit (N = 71, marginal R^2^ = 0.31)*				
**Playback treatment**	**-**	**12.71**	**2**	**< 0.001**
Conspecific vs heterospecific intra-habitat	0.40 ± 0.36	1.12	60	0.266
** Conspecific vs heterospecific extra-habitat**	**1.16 ± 0.34**	**3.39**	**60**	**0.004**
** Heterospecific intra-habitat vs heterospecific extra-habitat**	**0.76 ± 0.31**	**2.41**	**60**	**0.023**
Already singing	0.58 ± 0.34	3.02	1	0.082
Prog. trial	-0.26 ± 0.14	3.34	1	0.067
Minutes after sunrise	0.14 ± 0.13	1.77	1	0.277

*Crested tit (N = 68, marginal R^2^ = 0.38)*				
**Playback treatment**	**-**	**31.83**	**2**	**< 0.001**
** Conspecific vs heterospecific intra-habitat**	**1.65 ± 0.37**	**4.41**	**58**	**0.001**
** Conspecific vs heterospecific extra-habitat**	**2.65 ± 0.30**	**5.44**	**58**	**< 0.001**
Heterospecific intra-habitat vs heterospecific extra-habitat	-0.01 ± 0.31	-0.02	58	0.987
Already singing	0.33 ± 0.34	0.93	1	0.335
Prog. trial	-0.02 ± 0.14	0.01	1	0.905
Minutes after sunrise	-0.01 ± 0.12	0.01	1	0.949

*Coal tit (N = 56, marginal R^2^ = 0.55)*				
**Playback treatment**	**-**	**37.89**	**2**	**< 0.001**
** Conspecific vs heterospecific intra-habitat**	**2.69 ± 0.41**	**6.57**	**46**	**< 0.001**
** Conspecific vs heterospecific extra-habitat**	**2.31 ± 0.32**	**7.11**	**46**	**< 0.001**
Heterospecific intra-habitat vs heterospecific extra-habitat	-0.38 ± 0.38	-1.00	46	0.322
Already singing	0.80 ± 0.44	3.32	1	0.070
Prog. trial	-0.10 ± 0.15	0.46	1	0.495
Minutes after sunrise	-0.06 ± 0.14	0.16	1	0.690


Finally, differences in body mass and similarities in song between species pairs were not significantly correlated with the score of their aggressive responses towards each other (*r* < 0.10 and *P* > 0.05 in both cases—see Appendix 3 and 4).

## Discussion

Playback experiments clearly demonstrated that tit species were able to recognize and respond to each other’s songs. While all the species behaved more aggressively against a conspecific intruder playback stimulus, results highlighted extremely coherent patterns in the response to heterospecific stimuli within each group of species. All “broadleaf species” responded more strongly towards territorial songs by other species from the same habitat compared to those from different habitats, while this was not the case for “conifer species.” Additionally, different species expressed aggression through different combinations of behavioral responses, highlighting the multidimensional nature of territorial behavior.

In line with our expectations, in each species aggression was consistently higher towards conspecific playbacks than towards heterospecific playbacks. This result is readily attributable to stronger competition for resources among conspecific individuals, in particular for maintaining exclusive access to mating partners, besides nest cavities and food resources (Hartley and Shorrocks 2002).

The stronger territorial aggression towards other species from the same habitat compared to those from different habitats that we observed among “broadleaf species” may be attributed to intense interspecific competition for similar resources due to overlapping foraging and breeding habitats during the synchronous breeding season (Berlusconi et al. 2025). It is therefore likely that the spacing of breeding territories of “broadleaf species” (Berlusconi et al. 2025), may represent the direct effect of such competitive interactions. Indeed, interspecific territorial aggression typically results in exclusive territories defended against heterospecifics (Grether et al. 2013). Phylogenetically and ecologically similar species occupying adjacent territories can recognize and respond to the songs of heterospecific intruders they regularly encounter (Doutrelant et al. 2000a; Depino and Areta 2020; Drury et al. 2020). Such response to heterospecific songs is generally considered adaptive (Martin et al. 1996) and endures only under persisting competition for limited ecological resources shared among coexisting species (Polechová and Storch 2019; Drury et al. 2020). Conversely, the co-occurrence of “broadleaf species” with “conifer species” did not promote any interspecific territorial display, in accordance with the absence of shared ecological requirements among species of the two groups.

Although some authors (Doutrelant et al. 2000a; Doutrelant and Lambrechts 2001; Gorissen et al. 2006) have clearly demonstrated the ability of blue tits to discriminate between conspecific and great tits songs, interspecific territoriality has not been documented in tit species so far. However, it should be highlighted that interspecific territoriality is often facultative, influenced by the local context and breeding densities (Drury et al. 2020). This behavior can emerge within local communities in response to variation in both intra- and interspecific competition intensity (MacArthur 1958; Drury et al. 2020). Indeed, the study area possesses several unique characteristics possibly favoring these competitive dynamics. Firstly, it is a tapestry of various broadleaf tree patches that provide a diverse range of resources and microhabitats, enabling the coexistence of three tit species in large populations. Secondly, different forest management practices have resulted in a paucity of old trees, which typically contains natural cavities suitable for tit species (Ke et al. 2023), possibly forcing individuals to compete for this limited resource during the breeding season. Similarly, previous studies have suggested that under restrictive environmental conditions, species that exploit highly similar (or even identical) foraging and habitat resources are more likely to evolve interspecific territoriality as a stable behavioral strategy (thus leading to spatial segregation of territories), rather than character displacement, to facilitate coexistence (Grether et al. 2009, 2017; Arrizabalaga-Escudero et al. 2018; Kent and Sherry 2020).

In contrast, “conifer species” exhibited similar levels of aggressive responses toward species from both the same and different habitats. Consequently, the observed minimal overlap of their breeding territories in the study area (Berlusconi et al. 2025) may not be attributable to interspecific aggression. A plausible interpretation is that resource partitioning mechanisms emerged at an unexplored spatial scale, which might lead to a natural reduction in interspecific aggression. The spatial segregation of territories could be influenced by variation in the distribution of resources, highlighting the potential role of resource partitioning and microhabitat characteristics in shaping interspecific interactions (Robinson and Terborgh 1995). This interpretation is enforced by the presence of the more specialized crested tit (Berlusconi et al. 2022), whose preferred habitats are uncommon in the study area. In addition, the crested tit may excavate its own nesting cavity (Cramp and Perrins 1993), which may help to reduce competition for limiting nest cavities. Additionally, the lower number of species in this group (two against three “broadleaf species”), together with the relatively low breeding density in the study area (Berlusconi et al. 2025) may result in reduced competition and less aggressive interactions. Further analyses at a finer spatial scale, as well as studies on the diet of both adults and nestlings, are needed to better understand competition dynamics among tit species.

Body size is often considered an important factor influencing interspecific interactions, including aggression and territoriality, as larger individuals may have an advantage in physical contests (Peters 1983; Donadio and Buskirk 2006; Bonner 2024). Such a pattern may also emerge among species, with larger ones potentially being more aggressive, especially towards smaller ones (Morse 1974; Wojczulanis-Jakubas et al. 2015). These competitive dynamics have also been previously documented in tits (Alatalo and Moreno 1987; Krams 1996;  Doutrelant et al. 2000b; Doutrelant and Lambrechts 2001). However, this was not the case in our study system, suggesting that interspecific aggression is mostly driven by shared habitat and therefore competition for resources, irrespective of the body size of competing species, as occurs in other avian communities (Martin and Ghalambor 2014). Another possible confounding variable may be represented by the heterospecific song similarity, since birds can respond to the vocalizations of other species when sharing similarities in frequency range and structure with their own (Beckers et al. 2003; Kirschel et al. 2009). However, the study species did not exhibit increased aggression towards interspecific competitors displaying similar songs, suggesting that coexisting tits do effectively recognize each other’s song, and do not exhibit misleading responses (Azar and Bell 2016; Kleyn et al. 2021; Hunt et al. 2024).

In conclusion, our study reveals that certain species within the guild discriminate among heterospecifics. We propose that these interactions may arise from niche overlap and competition for space and resources, irrespective of body size and song similarities among interacting species, possibly also occurring in other regions across Europe where these species coexist. Ultimately, the current study contributes to our understanding of the mechanism regulating intra-guild interactions and interspecific coexistence, offering novel insights into the behavioral mechanisms that shape bird communities maintaining biodiversity in the long term.

## Supplementary Material

araf082_suppl_Supplementary_Materials_1

## Data Availability

Analyses reported in this article can be reproduced using the data provided by Berlusconi et al. (2025).
