# Supplementary material for: Heterospecific territorial defense in tit species varies according to breeding habitat overlap
Source: Behav Ecol. 2025 Jul 20;36(4):araf082. doi: 10.1093/beheco/araf082 (PMC12322488; doi:10.1093/beheco/araf082)
Supplement: araf082_suppl_Supplementary_Materials_1 [file araf082_suppl_supplementary_materials_1.zip › araf082_suppl_Supplementary_Tables_S1-S4_Figures_S1-S7.rtf]

Supplementary material for:

Heterospecific territorial defense in tit species varies according to breeding habitat overlap


List of contents:

Appendix 1: Comparison of breeding phenology among species.				Pag. 3
Table S1: Egg-laying dates for four tit species.						Pag. 3
Appendix 2: Territory mapping procedure.							Pag. 4
Figure S1: Map of identified male territories.						Pag. 6
Table S2: Scores for each species across playback treatments.				Pag. 7
Table S3: Scores for each combination among focal and playback species.		Pag. 8
Figure S2: Behavioral responses by species across playback treatments.			Pag. 9
Figure S3. Behavioral responses of great tit across playback treatments.			Pag. 10
Figure S4. Behavioral responses of blue tit across playback treatments.			Pag. 11
Figure S5. Behavioral responses of marsh tit across playback treatments.			Pag. 12
Figure S6. Behavioral responses of crested tit across playback treatments.		Pag. 13	
Figure S7. Behavioral responses of coal tit across playback treatments.			Pag. 14
Appendix 3: Correlation analysis between aggressiveness and body size.			Pag. 15
Appendix 4: Correlation analysis between aggressiveness and song similarity.		Pag. 16
Table S4: Matrix of song dissimilarity among species.					Pag. 18
References											Pag. 19


Appendix 1. Comparison of breeding phenology among species

A series of nest boxes (N = 198) has been installed within the Pineta Park of Appiano Gentile and Tradate – located outside the areas used for territory mapping. Nest boxes were randomly located throughout the wood areas. These nest boxes were monitored in order to obtain the phenological breeding parameters of the studied species in parallel with the experiments.  
Specifically, in 2023, we recorded the egg-laying dates for great tits (N = 35), blue tits (N = 23), marsh tits (N = 8), and coal tits (N = 15) (Table S4). Crested tits did not occupy any of the available nest boxes. Since egg-laying dates provide a reliable indicator of the breeding period, we used them to assess potential differences in reproductive timing among species. To assess potential differences in breeding phenology among species, we compared egg-laying dates using a one-way ANOVA. Egg-laying dates resulted to be very similar among the four tit species, with differences among average values of maximum 4 days (Table S4). The analysis revealed no statistically significant differences among species (df = 3, F3.75 = 1.67, P = 0.18), indicating that the timing of reproduction was largely overlapping. 

Table S1. Egg-laying dates for four tit species.

Average (± standard deviation) egg-laying dates for four tit species recorded in the study area. Dates are expressed as calendar days.

Species	Laying date (mean ± SD) 	
Great tit	13th April ± 7.45 days	
Blue tit	11th April ± 4.71 days	
Marsh tit	11th April ± 9.27 days	
Coal tit	15th April ± 4.71 days	


Appendix 2. Territory mapping procedure.

We identified the territories of breeding pairs by relying on “territory mapping” method of unmarked birds (Bibby et al. 2007). We selected four territory mapping areas to survey (mean ± SD size of 60.1 ± 10.8 ha, Fig. S1), ensuring that they encompassed diverse habitat classes with similar coverage, ensuring all species to co-occur, and being characterized by a dense network of paths, in order to facilitate comprehensive coverage of the entire area during data collection, as suggested by (Bibby et al. 2007). A total of 7 surveys were conducted in each area, starting on March 16th 2023 and concluding on April 12th 2023, the period corresponding to territory acquisition by males and pair formation in our study area (our unpublished data); we relied on this period to standardize results and aggressive answers. All surveys were carried out from sunrise (7:00-8:00 AM) until midday (11:00-12:00 AM). For each mapping area, surveys were spaced apart by a minimum of 3 days between each consecutive session. All observations were digitalized using QGIS version 2.18.28 (QGIS development team 2020), after georeferencing each position of individuals as points.
For each species, observations were assigned to putative male territories following the standard methodology outlined by (Bibby et al. 2007). This involved analyzing clusters of bird observations to determine which points belonged to individual territories. Specifically, clusters with multiple simultaneous registrations of individuals were essential for accurately identifying the points associated with males. Subsequently, bird records were visually assigned to the putative territories of male individuals.
At the end of the process, we defined a territory when accomplishing the following criteria outlined in (Berlusconi et al. 2024): a minimum of 5 points, collected from at least 2 separate surveys and a minimum of 1 behavior indicating the occurrence of a nest (e.g. territorial singing, entering a cavity, nest building, etc.) was observed. This procedure resulted to be more robust to identify territorial boundaries than the commonly used mapping method (Bibby et al., 2007). Then, the minimum convex polygon was used as a method to delimit territories (see Hill and Lein 1989; Assandri et al. 2018; Juárez et al. 2020), using “adehabitatHR” package (Calenge 2006). 
A total of 106 putative territories were identified and subsequently validated in the field during playback trials, specifically relaying on conspecific stimuli (see below). In 5 putative territories (only ~3.77% out of the total) males never responded (including conspecific stimuli), and thus they were excluded from the subsequent experiments. A total of 101 territories were then tested, consisting of 21 territories of great tit, 21 of blue tit, 21 of marsh tit, 20 of crested tit and 18 of coal tit (see Fig. S1 for the map of territories). Each territory received an identification code. 
The total number of territories recorded may appear lower than the average for tit species. However, this stems from the territory mapping procedure conducted at an early stage of breeding period (see above), which, although optimal for detectability, captured fewer territories than are actually present in the area. A recent study in the same sites (Berlusconi et al. 2024), carried out in 2022, documented nearly twice as many territories (N = 456) by extending the sampling period twice (two months). At the time we concluded this experiment, the number was similar to the count of territories recorded for the same dates in 2022.


Figure S1. Map of identified male territories. 
Territories of tit males identified during the mapping procedure which were subjected to playback experiments (see Methods), for the 4 mapping areas: A, G, C, E. The colors of each territory indicate the species to which males belong: yellow = great tit; blue = blue tit; purple = marsh tit; white = crested tit; black = coal tit. Each territory has an identification code. White lines indicate paths.


Table S2. Scores for each species across playback treatments.

Mean values of the total aggression score (± SE) for each species in each playback treatment. Scores were calculated based on the four behavioral variables considered (see “Quantifying territorial male responses to playback” in Methods). Numbers in parentheses indicate sample sizes (N). Values are expressed as mean value (bold) ± standard error; range is reported in square brackets [minimum – maximum]. Corresponding visual representation provided in Figure S2.

		Playback treatment	
		Conspecific	Heterospecific intra-habitat	Heterospecific extra-habitat	
Species	Great tit	2.39 ± 0.14 [1.41 – 3.50] (20)	1.48 ± 0.20 [0.00 – 2.70] (23)	0.99 ± 0.15 [0.00 – 2.52] (26)	
	Blue tit	2.32 ± 0.19 [0.00 – 3.42] (21)	1.49 ± 0.16 [0.00 – 2.62] (26)	0.88 ± 0.16 [0.00 – 2.47] (21)	
	Marsh tit	2.15 ± 0.20 [0.16 – 3.29] (20)	1.81 ± 0.23 [0.00 – 2.81] (22)	1.20 ± 0.18 [0.00 – 3.00] (26)	
	Crested tit	1.93 ± 0.21 [0.00 – 3.04] (18)	0.70 ± 0.23 [0.00 – 2.80] (15)	0.89 ± 0.17 [0.00 – 3.21] (33)	
	Coal tit	2.36 ± 0.25 [0.00 – 3.65] (18)	0.70 ± 0.30 [0.00 – 2.60] (11)	0.67 ± 0.19 [0.00 – 2.88] (27)	


Table S3. Scores for each combination among focal and playback species.

Mean values of the total aggression score (± SE) for each combination of focal and playback species. Scores were calculated based on the four behavioral variables considered (see “Quantifying territorial male responses to playback” in Methods). Numbers in parentheses indicate sample sizes (N). Cell colors indicate habitat associations: great tit, blue tit, and marsh tit are “broadleaf species” (light green), while crested tit and coal tit are “conifer species” (light blue).

		
	Playback species	
	 	Great tit	Blue tit	Marsh tit	Crested tit	Coal tit	
Focal species	Great tit	2.39 ± 0.14 (20)	1.80 ± 0.25 (12)	1.13 ± 0.29 (11)	0.96 ± 0.24 (12)	1.01 ± 0.20 (14)	
	Blue tit	1.67 ± 0.21 (14)	2.32 ± 0.19 (21)	1.29 ± 0.25 (12)	0.53 ± 0.17 (9)	1.16 ± 0.23 (12)	
	Marsh tit	1.73 ± 0.32 (11)	1.74 ± 0.34 (11)	2.15 ± 0.20 (12)	1.23 ± 0.24 (18)	1.28 ± 0.27 (14)	
	Crested tit	0.80 ± 0.30 (11)	1.07 ± 0.31 (11)	0.72 ± 0.27 (12)	1.93 ± 0.21 (18)	0.75 ± 0.25 (14)	
	Coal tit	0.50 ± 0.22 (9)	0.93 ± 0.39 (10)	0.52 ± 0.35 (8)	0.70 ± 0.30 (11)	2.36 ± 0.25 (18)	


Figure S2. Behavioral responses by species across playback treatments.

Behavioral responses of territorial males across playback treatments. The panels show minimum distance to the speaker, mean distance to the speaker during singing, total time spent singing, and latency of the vocal response to playback for each species in response to conspecific, heterospecific intra-habitat, and heterospecific extra-habitat stimuli. Dots represent average values, while whiskers the standard errors. The colors indicate the species: yellow = great tit; blue = blue tit; purple = marsh tit; white = crested tit; black = coal tit.


Figure S3. Behavioral responses of great tit across playback treatments.
Fitted values from 4 separate Linear Mixed Models assessing the behavioral responses of territorial great tits to different playback treatments. Each model included one of the following behavioral variables as the dependent variable: (1) minimum distance to the speaker, (2) mean distance to the speaker while singing, (3) total time spent singing, and (4) latency of the vocal response. In all models, playback treatment was included as a fixed effect, with the following test statistics respectively: (1): ÷² = 40.78, P < 0.001; (2): ÷² = 43.10, P < 0.001; (3): ÷² = 13.08, P = 0.001; (4): ÷² = 17.79, P < 0.001. Dots represent model-predicted means; whiskers indicate standard errors. Asterisks denote statistically significant differences between treatments in post-hoc tests (P < 0.05; P-values corrected using the False Discovery Rate).


Figure S4. Behavioral responses of blue tit across playback treatments.
Fitted values from 4 separate Linear Mixed Models assessing the behavioral responses of territorial blue tits to different playback treatments. Each model included one of the following behavioral variables as the dependent variable: (1) minimum distance to the speaker, (2) mean distance to the speaker while singing, (3) total time spent singing, and (4) latency of the vocal response. In all models, playback treatment was included as a fixed effect, with the following test statistics respectively: (1): ÷² = 36.99, P < 0.001; (2): ÷² = 45.60, P < 0.001; (3): ÷² = 11.30, P = 0.003; (4): ÷² = 16.61, P < 0.001. Dots represent model-predicted means; whiskers indicate standard errors. Asterisks denote statistically significant differences between treatments in post-hoc tests (P < 0.05; P-values corrected using the False Discovery Rate).


Figure S5. Behavioral responses of marsh tit across playback treatments.
Fitted values from 4 separate Linear Mixed Models assessing the behavioral responses of territorial marsh tits to different playback treatments. Each model included one of the following behavioral variables as the dependent variable: (1) minimum distance to the speaker, (2) mean distance to the speaker while singing, (3) total time spent singing, and (4) latency of the vocal response. In all models, playback treatment was included as a fixed effect, with the following test statistics respectively: (1): ÷² = 18.61, P < 0.001; (2): ÷² = 11.04, P = 0.004; (3): ÷² = 14.97, P < 0.001; (4): ÷² = 0.97, P = 0.61. Dots represent model-predicted means; whiskers indicate standard errors. Asterisks denote statistically significant differences between treatments in post-hoc tests (P < 0.05; P-values corrected using the False Discovery Rate).


Figure S6. Behavioral responses of crested tit across playback treatments.
Fitted values from 4 separate Linear Mixed Models assessing the behavioral responses of territorial crested tits to different playback treatments. Each model included one of the following behavioral variables as the dependent variable: (1) minimum distance to the speaker, (2) mean distance to the speaker while singing, (3) total time spent singing, and (4) latency of the vocal response. In all models, playback treatment was included as a fixed effect, with the following test statistics respectively: (1): ÷² = 18.88, P < 0.001; (2): ÷² = 11.03, P = 0.004; (3): ÷² = 46.14, P < 0.001; (4): ÷² = 16.34, P < 0.001. Dots represent model-predicted means; whiskers indicate standard errors. Asterisks denote statistically significant differences between treatments in post-hoc tests (P < 0.05; P-values corrected using the False Discovery Rate).


Figure S7. Behavioral responses of coal tit across playback treatments.
Fitted values from 4 separate Linear Mixed Models assessing the behavioral responses of territorial coal tits to different playback treatments. Each model included one of the following behavioral variables as the dependent variable: (1) minimum distance to the speaker, (2) mean distance to the speaker while singing, (3) total time spent singing, and (4) latency of the vocal response. In all models, playback treatment was included as a fixed effect, with the following test statistics respectively: (1): ÷² = 59.17, P < 0.001; (2): ÷² = 40.10, P < 0.001; (3): ÷² = 40.51, P < 0.001; (4): ÷² = 58.66, P < 0.001. Dots represent model-predicted means; whiskers indicate standard errors. Asterisks denote statistically significant differences between treatments in post-hoc tests (P < 0.05; P-values corrected using the False Discovery Rate).

Appendix 3. Correlation analysis between aggressiveness and body size.	

Body size is typically considered an important factor influencing interspecific dominance and territoriality, since larger individuals may have an advantage in physical aggressions (Donadio and Buskirk 2006; Wojczulanis-Jakubas et al. 2015; Bonner 2024). Tits vary in body size and aggressiveness, so it is possible that smaller species, in general, will respond less aggressively to larger ones and vice versa (Alatalo and Moreno 1987; Krams 1996). To examine for this possible confounding effect, we assessed the correlation between interspecific aggression and body mass differences among species.
First, we gathered body mass data for the tit species. We used records from ringed individuals in the ANITA databank (Italian Bird Ringing Centre, ISPRA), selecting observations from adult males from Northern Italy (excluding the Alps). Great tits had an average body mass of 16.77 g (N = 6707), blue tits 10.42 g (N = 1896), marsh tits 10.06 g (N = 148), crested tits 11.53 g (N = 26) and coal tits 8.87 g (N = 40). Using these values, we constructed a matrix of body mass differences among species: for each pairwise species comparison, we calculated the absolute difference in mean body mass. This matrix was then used to assess whether differences in body size correlated with aggression scores observed for each combination of focal and playback species (i.e.: those reported in Table S3).
We performed a correlation analysis between the two matrices to assess their association. Since diagonal values represented conspecific interactions, which were not relevant for evaluating whether body mass differences mediate heterospecific aggression, we excluded them from the analysis. To determine statistical significance, we used a permutation-based approach in which all possible permutations of rows and columns were systematically generated and applied to the matrix, ensuring that each unique configuration was used only once (resulting in 120 permutations in our case). Only the non-diagonal values were considered in the analysis, as they represent heterospecific interactions. For each permutation, we recalculated the Pearson correlation coefficient, generating a null distribution. The observed correlation was then compared to this null distribution to compute a P-value, representing the probability of obtaining the observed correlation by chance. Results suggested that differences in body mass among species do not significantly correlate with their levels of aggressiveness (r = 0.07, P = 0.617).


Appendix 4. Correlation analysis between aggressiveness and song similarity.	

Birds can respond to the vocalizations of unfamiliar species, particularly when these share similarities in frequency range and structure with their own (Beckers et al. 2003; Kirschel et al. 2009). This is because many species have evolved the ability to recognize and respond to heterospecific calls, as such vocalizations carrying crucial information as danger or alarms (Fallow et al. 2011; Dutour et al. 2020; Sandoval and Wilson 2022). According to this, acoustically similar species may respond more aggressively to specific playback stimuli used in the experiment that closely resemble their own song characteristics. Thus, we assessed the correlation between interspecific aggression and song similarity among species.
We relied on songs selected for playback stimuli (see Methods in the main text for details). From each of the three recordings per tit species, we extracted a single phrase. These phrases were high-pass filtered at 100 Hz, normalized, and modified with a 20 ms fade-in and fade-out. Using the Dynamic Time Warping (DTW) algorithm in the Luscinia sound software (Lachlan 2022), we compared all phrases against each other and extracted dissimilarity measures for each species pair. DTW works by aligning syllables of phrases, based on the trajectories of a series of acoustic features (see Zandberg et al. 2024): fundamental frequency and mean frequency (log-transformed), fundamental and mean frequency change (the arcsin transform of the slope of these features on the spectrogram), normalized fundamental frequency (where the syllable-wide mean of fundamental frequency is subtracted from the fundamental frequency), and vibrato amplitude (a measure of sinusoidal signal in the fundamental frequency). Finally, time itself is included as a feature in order to penalize time warping. When two phrases are compared using this DTW process, a Euclidean distance is calculated over each of these features for each point in one syllable compared with each point in the other syllable. A dynamic algorithm then searches for an efficient alignment between the two syllables using these distances, and an overall dissimilarity is then calculated by averaging dissimilarities over the alignment of the entire phrases. Based on these dissimilarity measures, we constructed a distance matrix (Table S6), which was then used to evaluate whether song similarity differences correlated with interspecific aggression scores for each focal and playback species combination (i.e. those reported in Table S3).
We then performed a correlation analysis between the two matrices, as for the correlation analysis between body size differences and interspecific aggression. We relied on the same permutation-based procedure described in Appendix 3. Results suggested that similarities in songs among species do not significantly correlate with their levels of aggressiveness (r = 0.09, P = 0.583). 


Table S4. Matrix of song dissimilarity among species.

For each pairwise species song comparison, we calculated dissimilarity measures relying on Dynamic Time Warping (DTW) algorithm in the Luscinia sound software (see Appendix 4 for details).

	
 	Great tit	Blue tit	Marsh tit	Crested tit	Coal tit	
Great tit	0.154	0.538	0.241	0.548	0.338	
Blue tit	0.538	0.224	0.506	0.486	0.382	
Marsh tit	0.241	0.506	0.124	0.494	0.327	
Crested tit	0.548	0.486	0.494	0.240	0.437	
Coal tit	0.338	0.382	0.327	0.437	0.189	


References

Alatalo RV, Moreno J. 1987. Body Size, Interspecific Interactions, and Use of Foraging Sites in Tits (Paridae). Ecology. 68(6):1773–1777. doi:10.2307/1939868.
Assandri G, Bogliani G, Pedrini P, Brambilla M. 2018. Beautiful agricultural landscapes promote cultural ecosystem services and biodiversity conservation. Agriculture, Ecosystems & Environment. 256:200–210. doi:10.1016/j.agee.2018.01.012.
Beckers GJL, Goossens BMA, Ten Cate C. 2003. Perceptual salience of acoustic differences between conspecific and allospecific vocalizations in African collared-doves. Animal Behaviour. 65(3):605–614. doi:10.1006/anbe.2003.2080.
Berlusconi A, Castiglione G, Wauters LA, Martinoli Alessio, Clerici E, Mologni A, Morganti M, Martinoli Adriano, Romano A, Rubolini D. 2024. Habitat partitioning and spatial segregation at multiple scales promotes year-round coexistence in a guild of forest songbirds. doi:10.1101/2024.08.26.609673. [accessed 2024 Nov 11]. http://biorxiv.org/lookup/doi/10.1101/2024.08.26.609673.
Bibby CJ, Burgess ND, Hill DA, Mustoe S. 2007. Bird census techniques. Academic Press, London.
Bonner JT. 2024. Why Size Matters: From Bacteria to Blue Whales. Princeton: Princeton University Press (Princeton Science Library).
Calenge C. 2006. The package “adehabitat” for the R software: A tool for the analysis of space and habitat use by animals. Ecological Modelling. 197(3–4):516–519. doi:10.1016/j.ecolmodel.2006.03.017.
Donadio E, Buskirk SW. 2006. Diet, Morphology, and Interspecific Killing in Carnivora. The American Naturalist. 167(4):524–536. doi:10.1086/501033.
Dutour M, Suzuki TN, Wheatcroft D. 2020. Great tit responses to the calls of an unfamiliar species suggest conserved perception of call ordering. Behav Ecol Sociobiol. 74(3):37. doi:10.1007/s00265-020-2820-7.
Fallow PM, Gardner JL, Magrath RD. 2011. Sound familiar? Acoustic similarity provokes responses to unfamiliar heterospecific alarm calls. Behavioral Ecology. 22(2):401–410. doi:10.1093/beheco/arq221.
Hill BG, Lein MR. 1989. Territory Overlap and Habitat Use of Sympatric Chickadees. The Auk. 106(2):259–268.
Juárez R, Chacón-Madrigal E, Sandoval L. 2020. Urbanization has opposite effects on the territory size of two passerine birds. Avian Res. 11(1):11. doi:10.1186/s40657-020-00198-6.
Kirschel ANG, Blumstein DT, Smith TB. 2009. Character displacement of song and morphology in African tinkerbirds. Proc Natl Acad Sci USA. 106(20):8256–8261. doi:10.1073/pnas.0810124106.
Krams IA. 1996. Predation Risk and Shifts of Foraging Sites in Mixed Willow and Crested Tit Flocks. Journal of Avian Biology. 27(2):153. doi:10.2307/3677144.
Lachlan RF. Luscinia: Bioacoustic analysis of field recordings (version 2.22.12.01.01) [Computer software] 2022, https://github.com/rflachlan/Luscinia/.
Sandoval L, Wilson DR. 2022. Neotropical Birds Respond Innately to Unfamiliar Acoustic Signals. The American Naturalist. 200(3):419–434. doi:10.1086/720441.
Wojczulanis-Jakubas K, Kulpiñska M, Minias P. 2015. Who bullies whom at a garden feeder? Interspecific agonistic interactions of small passerines during a cold winter. J Ethol. 33(2):159–163. doi:10.1007/s10164-015-0424-x.
Zandberg L, Morfi V, George JM, Clayton DF, Stowell D, Lachlan RF. 2024. Bird song comparison using deep learning trained from avian perceptual judgments. Nogueira W, editor. PLoS Comput Biol. 20(8):e1012329. doi:10.1371/journal.pcbi.1012329.
